# A histopathological snapshot of bladder cancer: a Johannesburg experience of 1480 histopathology reports

**DOI:** 10.1007/s00345-025-05540-5

**Published:** 2025-03-11

**Authors:** Jaclyn Jonosky, Ahmed Adam, Reubina Wadee

**Affiliations:** 1 Division of Urology, Department of Surgery, University of the Witwatersrand, Johannesburg, South Africa; 2 Department of Anatomical Pathology, University of the Witwatersrand, /National Health Laboratory Services, Johannesburg, South Africa; 3 Charlotte Maxeke Johannesburg Academic Hospital, 17 Jubilee Road, Johannesburg, Parktown, 2193 South Africa

**Keywords:** Bladder cancer, Urothelial carcinoma, Histopathology, Johannesburg, Squamous cell carcinoma, Non-muscle invasive bladder cancer, Muscle-invasive bladder cancer

## Abstract

**Purpose:**

To evaluate the histopathological characteristics of bladder cancer in patients presenting to Johannesburg hospitals over a 13-year period (2010–2023).

**Methods:**

Following ethical clearance, a retrospective observational, descriptive review of histopathological reports over 13 years was conducted in Johannesburg. Inclusion criteria was bladder biopsies, TURBT specimens, and radical cystectomy (RC) specimens positive for bladder cancer. Exclusion criteria was non-primary bladder cancers (prostate, cervical, colon) and urothelial carcinoma of upper tract origin (*N* = 970). Of the initial specimens (*N* = 2450), 1480 met the inclusion criteria, representing 858 patients, owing to multiple transurethral resections of bladder tumours (TURBT). Categorical variables were summarised as counts and percentages, while numerical variables were reported as means with standard deviations or medians with interquartile ranges, depending on data distribution and tested via the Shapiro‒Wilk test. Statistical comparisons were performed using Fisher’s exact test (sex), one-way ANOVA, or the Kruskal‒Wallis test (age). Statistical significance was set at *p* < 0.05.

**Results:**

Urothelial carcinoma accounted for 88.8% of bladder cancer, squamous cell carcinoma (7.7%), adenocarcinoma (1.5%), and other malignancies (2%). High-grade urothelial carcinoma was predominant at 75%. Non-muscle invasive disease accounted for 72% of these cases, while 28% were muscle invasive. Data from radical cystectomies showed a high proportion of aggressive and advanced disease.

**Conclusions:**

The study highlights the predominance of high-grade non-muscle invasive bladder cancer in Johannesburg, consistent with global trends. The findings suggest a shift in bladder cancer trends in Johannesburg away from assumed squamous cell carcinoma towards urothelial carcinoma.

## Introduction

Bladder cancer (BC) is the 9th most common malignancy and the 13th most common cause of death globally according to the GLOBOCON statistics of 2022 [1–3]. It ranks as the fourth most common malignancy in males and the 11th most common malignancy in females worldwide [4]. According to the most recent data from the South African National Cancer Registry (NCR), bladder cancer accounted for 959 new cases in males (2.29%) and 340 new cases in females (0.74%) in 2022 [6].Urothelial carcinoma (UC) is the most prevalent subtype with non-muscle invasive bladder cancer (NMIBC) accounting for 70–75% of cases [2, 3] while muscle-invasive bladder cancer (MIBC) accounting for the remainder [2, 3]. NMIBC, though associated with low mortality, has high recurrence rates (70%) and a 45% progression rate within five years for high-risk cases [3, 7].

Smoking and occupational exposures are leading risk factors for UC in developed nations, while schistosomiasis and chronic bladder irritation predominate as risk factors for SCC in developing countries [5]. Urbanization in developing regions has shifted patterns, with increasing UC incidence [7]. Smoking prevalence globally is 19%, while South Africa reported 24% in 2021 [8]. Johannesburg, with a population exceeding six million, represents an economic and employment hub, attracting patients from surrounding regions [9].

Data on BC in South Africa, is limited [5]. Although it is reported as one of the leading causes of cancer in Africa, with an estimated incidence of 7.1 per 100,000, the available data is sparse and outdated [2, 10] as shown in Table 1 below. Table 1 shows a literature review of data available in African countries.

**Table 1:** Tabulation of previous published data on bladder Cancer from the continent of Africa

Author	Publication Date	Type of Study	Units of Patients/Studies/samples	Country	Purpose of Study	Key Findings of the Study
Adeloye et al.	2019	Systematic Review	22 Studies	15 African countries	Epidemiology of Bladder Cancer	Rates of bladder cancer increasing
Bowa et al.	2018	Systematic Review	23 Studies	Sub-Saharan Africa	Epidemiology of Bladder Cancer	SCC Bladder Predominant
Cassel et al.	2019	Systematic Review	47 Studies	10 African countries	Epidemiology of Bladder Cancer	SCC Predominant However UC increasing
Groeneveld et al.	1996	Retrospective Review	615 Patients	KZN, South Africa	Histological Variations in Racial Groups	SCC in African patients, UC in Caucasian patients
Saouli et al.	2021	Retrospective Review	39 Patients	Morocco	Histological Variants in Bladder Cancer	Predominance of Squamous and micropapillary differentiation
Ssekitooleko et al.	2024	Descriptive Cross-Sectional Study	117 Samples	Uganda	Epidemiology of Bladder Cancer	UC most common finding in bladder cancer
Yohana et al.	2023	Retrospective Review	481 Patients	Tanzania	Trend of SCC Bladder	SCC most common, increasing numbers of UC

## Materials and methods

Following ethical approval (clearance number: M231189), a retrospective review of histopathological reports (*N* = 2450) from 2010 to 2023 was conducted. The hospital is an academic center and provides pathology services to nine peripheral hospitals. A total number of 2450 specimens were initially reviewed, 1480 samples were appropriate for analysis. A total of 858 patients were included in the study, many of these patients received multiple trans-urethral resection of bladder tumours (TURBT) therefore total number of relevant histological reports was 1480. Inclusion Criteria was all positive bladder cancer pathology reports; bladder biopsies positive for bladder cancer, TURBT specimens and radical cystectomy (RC) specimens.

Exclusion Criteria were submitted cancer reports that were not primary bladder cancer (*N* = 970). This included pathology reports that were positive for cervical carcinoma, colon carcinoma, prostate adenocarcinoma. Bladder biopsies that were negative for malignancy were also excluded unless prior TURBT’s showed malignancy. In addition, urothelial carcinoma of upper tract origin was excluded.

### Statistical analysis

An observational, descriptive study of histopathological reports was performed. The data was analyzed using IBM SPSS statistics software, version 29. The patient demographics and clinical profiles are shown as counts (N) and percentages (%) for categorical variables. For numerical variables, the results are reported as the means with standard deviations (for normally distributed data) and as medians with interquartile ranges (for nonnormally distributed data). The normality of the distribution was tested via the Shapiro‒Wilk test. Histopathological results were compared by patient sex via Fisher’s exact test as well as by patient age via either one-way ANOVA or the Kruskal‒Wallis test (for nonnormally distributed data). All the results of all the statistical analyses were considered significant at p values < 0.05.

## Results

In terms of patient demographics, our study revealed that 75% (*N* = 644) of patients diagnosed with bladder cancer were male and 25% (*N* = 214) were female. The mean age at presentation was 62 years. The youngest patient in our study was 4 years old (who had rhabdomyosarcoma of the bladder), and the oldest was 94 years old, with a standard deviation of 13.1 years. In our study, 88% of patients (*N* = 760) underwent TURBTs, whether single or repeat procedures, whereas 11.4% (*N* = 98) underwent radical cystectomy.

**Table 2:** Tabulation of histological subtypes and demographics of bladder Cancer in current study

		Demographics		
Histological Sub-type	Percentage Distribution in Current Study (%)	Age (Years)	Sex (%)	
			M	F
Urothelial Carcinoma	88	64	79	21
Squamous Cell Carcinoma	7.7	52	41	59
Adenocarcinoma	1.5	57	61	39
Other Malignancies	2.0	46	50	50

As shown in Table 2 above, UC accounted for 88% of cases (*N* = 762). SCC was 7.7% (*N* = 66) and primary bladder adenocarcinoma (AC) was 1.5% (*N* = 13). Other primary bladder malignancies accounted for 2% of tumors (*N* = 17).

With respect to UC, 88% (*N* = 674) of our samples were from TURBTS, whereas 12% (*N* = 88) were from RC. Males accounted for 79%, while females were 21%. The mean age at diagnosis was 64 years.

**Fig. 1:**
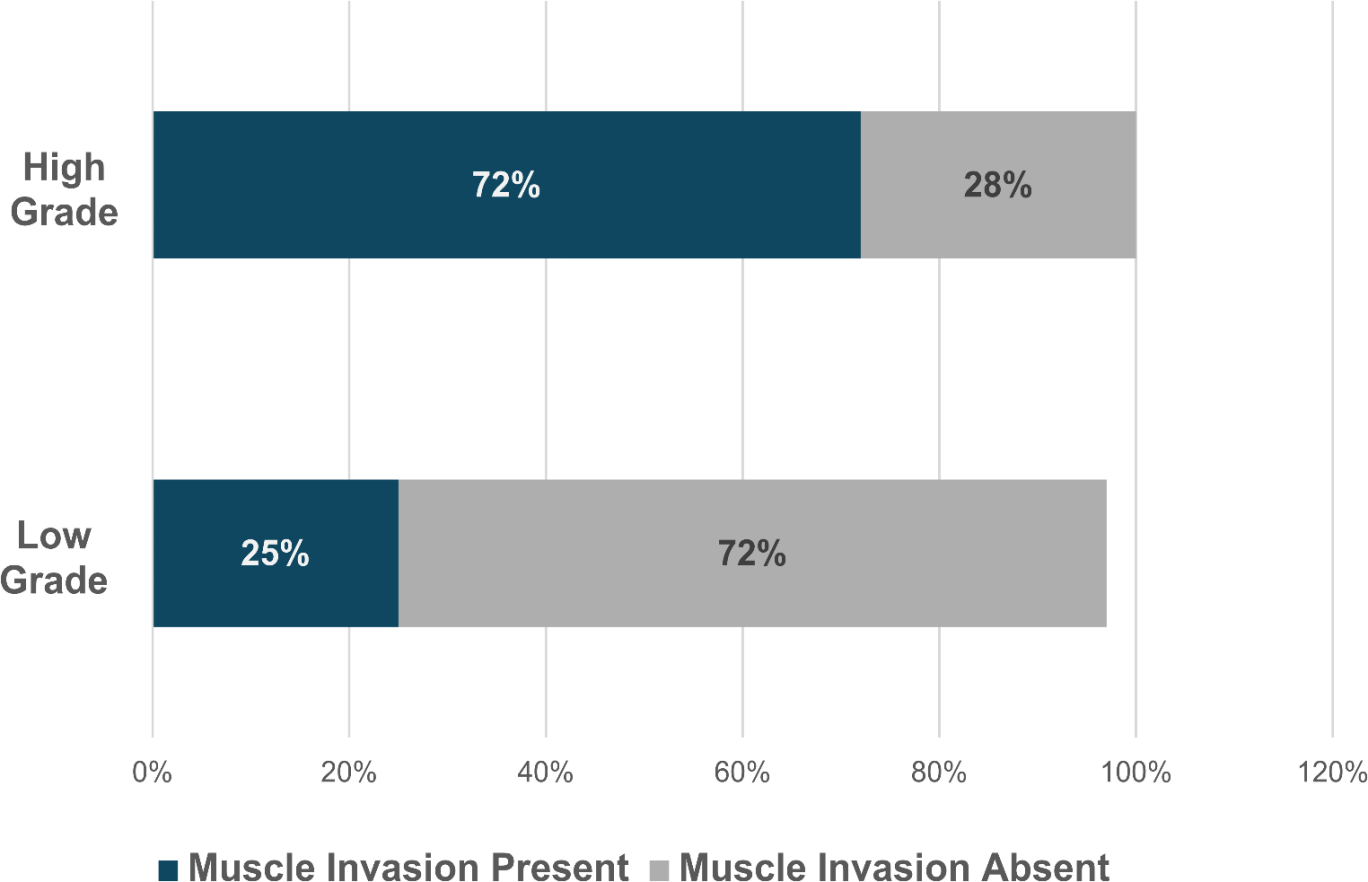
Urothelial carcinoma: Histological Profile according to Grade of Disease and Muscle Invasion

High-grade UC was found in 75% of patients (*N* = 572), whereas low-grade urothelial carcinoma was found in 25% (*N* = 190). Carcinoma in situ (CIS) was found in 7.3% (*N* = 56). NMIBC accounted for 72% (*N* = 535) of cases, whereas MIBC was found in 28% (*N* = 250). Figure 1 shows the proportion of high grade versus low grade disease as well as proportion of muscle invasiveness in each category.

Regarding TURBT data for UC, 27% of the samples were low grade, whereas 73% were high grade. Detrusor muscle was represented 43% of the cases in our dataset, and muscle invasion was present in 23% of cases. The remainder of patients were diagnosed with NMIBC. Stage Ta accounted for 41% of cases, while T1 accounted for 35%. CIS was found in 6%, and lymphovascular invasion (LVI) was identified in 1.8%. Squamous differentiation was found in 8%, cystitis cystica in 2%, and cystitis glandularis was found in 0.4%. Von Brunn’s nests, follicular cystitis and adenomatous differentiation were observed in 0.1%. We assessed the clinical characteristics by sex, and our study revealed no significant difference between the grade of tumors and NMIBC versus MIBC between males and females (p value < 0.5).

Among the RCs performed for UC, 87% of the samples had high-grade disease, and MIBC accounted for 77%. The remainder were for NMIBC, and only 12% of these were low-grade specimens. CIS was found in 15% of the RC samples. LVI was found in 37% in this group. Squamous differentiation was observed in 5%, glandular differentiation in 1%, and sarcomatoid features in 6%. Cystitis cystica was found in 4% of these cases, and cystitis glandularis was observed in 1%. With respect to the involvement of local structures, the prostate was involved in 15% of cases, the lymph nodes were positive in 29%, ureteric involvement was observed in 7%, and seminal vesicles were observed in 5%. Carcinoma was upstaged from TURBT in 35% of the patients. Incidental prostate adenocarcinoma was found in 10% in our current study, with Gleason 3 + 3 (WHO Grade Group 1) carcinomas being predominant.

Our study revealed SCC in 7% of the patients. TURBTs accounted for 88% (*N* = 58) of the cases in this group, whereas RC specimens accounted for 12% (*N* = 8). The mean age at diagnosis for SCC patients was 52 years; males accounted for 41% (*N* = 27) of the patients, whereas females accounted for 59% (*N* = 39). Schistosomiasis was present in 38% of the samples. The limited number of RCs included advanced-stage disease (T2b-12%, T3b-25%, and T4-62% of cases).

Primary bladder adenocarcinoma was found in less than 2% of specimens (*N* = 13). Other malignancies accounted for 2% of cases and included spindle cell sarcoma, neuroendocrine tumors, rhabdomyosarcomas, lymphomas and clear cell carcinoma of the Mullerian type.

## Discussion

This study provides valuable insights into the histopathological patterns of BC in Johannesburg, South Africa, and addresses a significant gap in the literature. Our findings indicate a predominance of BC in male patients, which is consistent with studies reporting that it is up to four times more common in males [3]. This gender disparity is often attributed to delayed diagnosis, differing help-seeking behaviors, and increased exposure to smoking and occupational hazards in males [2].

Our study confirms that UC is the most prevalent form of cancer, which aligns with both international and limited African studies [2–4]. Historically SCC has been the dominant histopathological subtype of BC in African countries because of the high incidences of schistosomiasis and schistosomal cystitis [5–7, 11]. However, with increasing urbanization and efforts to control schistosomiasis, the incidence of UC is increasing, particularly in nonendemic areas such as Johannesburg [4, 7, 11].

The study also revealed a predominance of NMIBC, which is consistent with global trends, where 75–80% of UC cases are reported as NMIBC [2]. Limited African data corroborate this finding, with NMIBC occurring in 75–85% of cases [4]. A previous study from 1996 indicated that 76% of UC cases in South Africa were staged as T1 tumors, which mirrors our current findings [7].

This study highlighted associations between certain pathological findings and clinical outcomes. Our study revealed CIS in 6% of TURBTs, whereas the presence of CIS at TURBT has been reported with a wide incidence range of 24–59% [14]. This variability is likely due to sampling errors and the positioning of the CIS relative to the tumor, prompting some authors to advocate for random bladder sampling, enhanced cystoscopy, and urine cytology to increase the detection of the CIS [11, 14]. Histopathological variants of UC, including squamous and glandular differentiation, micropapillary, plasmacytoid, sarcomatoid, and nested variants, have been reported in 25% of cases and are generally associated with a more aggressive clinical course [3, 12]. In our study, squamous differentiation was observed in 8% of the TURBT samples and 5% of the RC samples. Squamous differentiation is linked to faster disease progression and higher recurrence rates; hence, rapid definitive treatment is recommended when it is detected [2]. Glandular differentiation, reported to occur in 18% of cases, is associated with poorer prognosis and was found in 1% of our radical cystectomy cohort [12].

RC was primarily performed for MIBC, with 87% of these specimens found to have high-grade disease. CIS at RCs is associated with higher recurrence rates, and our study revealed that the incidence of CIS at RCs was 15%, lower than that reported in the literature but higher than our TURBT findings [3, 13]. Lymph node involvement, which was present in 29% of the patients in our study, remains a critical prognostic factor, with an increasing number of positive nodes correlated with increased recurrence risk [11]. Additionally, 35% of carcinoma cases at RCs are upstaged from the original TURBT, which has been associated with higher rates of additional lymph node involvement and impacts recurrence and overall survival [15].

Incidental prostate adenocarcinoma was found in 10% of cases, predominantly with a combined Gleason score of 6, which equates to WHO grade group 1. This aligns with reported rates of incidental prostate carcinoma at RCs, which range between 24% and 51% [16]. Most incidental prostate cancer cases are organ confined and clinically insignificant, possibly reflecting the high incidence of both cancers in an aging population [16, 17].

Bladder SCC accounted for nearly 7% of the cases, with most samples being TURBTs and very few RCs being performed. This is likely because 27% of patients present with advanced-stage disease at the outset, rendering many patients inoperable at diagnosis [4, 7, 11]. SCC is often muscle invasive and has an advanced stage of disease (T3/T4) at initial presentation, accounting for approximately 90% of cases [7, 11]. Our study revealed that SCC patients were diagnosed at a younger age than UC patients were, which aligns with reported trends [7]. However, we observed a female predominance (59%), whereas the literature typically reports a male predominance [7]. Schistosomiasis was detected in 38% of the SCC patients in our study, with reports in a limited number of African studies ranging from 45 to 85% [7]. RC for SCC shows a predominance of higher-stage disease, which is consistent with the literature indicating that most SCC are muscle invasive at diagnosis, potentially due to differences in pathogenesis and tumor spread [7, 11].

Our study revealed primary bladder adenocarcinoma in 1.5% of the samples. Primary bladder adenocarcinoma is staged higher than UC at diagnosis, with the involvement of local structures, which is consistent with the limited literature on this clinical entity [11]. Other malignancies were observed only in a small percentage of our cohort. Patients presenting with these malignancies often face challenges due to their aggressive nature and poor prognosis [11]. Furthermore, treatment options for these malignancies are controversial and widely debated owing to the small number of guidelines and treatment algorithms. For such patients, the trend is toward early RC [11].

## Conclusion

This study evaluated the histopathological features of bladder cancer in Johannesburg over 13 years and revealed a predominance of high-grade NMIBC. RC performed for muscle-invasive disease revealed histologically advanced disease with significant lymph node involvement, likely due to the predominance of high-grade UC. Our study revealed low levels of SCC, possibly because Johannesburg is not an endemic bilharzia area, although the SCC cases observed in our study were found to have relatively high pathological stages.

Our findings suggest a shift in bladder cancer trends in Johannesburg away from SCC toward UC. The large cohort of patients in the NMIBC group reflects the high morbidity of bladder cancer, the likely high return of patients to the health care system repeatedly, high repeat procedure rates and risk of progression of their malignancy. Although no conclusions could be drawn in the current study due to the retrospective nature and review of pathology reports, as to the association between smoking and UC, smoking is known to be the greatest risk factor for urothelial bladder cancer. Based on this reported information, a better anti-smoking campaign and more effective prevention strategies are recommended. Continued efforts toward the treatment and screening of Bilharzia are equally imperative owing to the advanced clinical presentation of these patients.

## Strengths and weaknesses of the study

The strengths of this study include its large number of urothelial carcinoma TURBT data. The current study has its limitations in regard to the retrospective nature of the study. This introduces biases, in terms of missing or incomplete data and interpretation. An additional limitation of the study is that no prospective or follow up data was obtainable and therefore causal interpretation and outcomes could not be established in the patient population being studied. Further, due to the study being a review of histopathological reports where the submitted clinical patient history is limited, we were unable to determine socio-economic and social factors that impact on various cancers reported as well as being unable to draw clear links to causal factors such as smoking.

## Data Availability

Data is provided within the manuscript.
